# Investigation of thermal changes in the thyroid gland region of individuals with hypothyroidism and fibromyalgia by analyzing the temperature of brown adipose tissue

**DOI:** 10.1038/s41598-021-85974-0

**Published:** 2021-03-22

**Authors:** Ana Paula Christakis Costa, Joaquim Miguel Maia, Marcos Leal Brioschi, José Eduardo de Melo Mafra Machado

**Affiliations:** 1 Graduate School of Electrical Engineering and Applied Computer Sciences (CPGEI), Federal University of Technology - Paraná (UTFPR), Avenida Sete de Setembro, 3165, Rebouças, Curitiba, Paraná 80230-901 Brazil; 2 Electronic Engineering Department and Graduate School of Electrical Engineering and Applied Computer Sciences (DAELN - CPGEI), Federal University of Technology - Paraná (UTFPR), Curitiba, Brazil; 3 Neurology Department of Clinic Hospital of São Paulo, University School of Medicine, Brazilian Association of Medical Thermology, São Paulo, SP Brazil; 4 Brazilian Association of Medical Thermology, São Paulo, SP Brazil

**Keywords:** Rheumatology, Biomedical engineering

## Abstract

This exploratory retrospective study aims to investigate the thermal changes in the thyroid gland region of patients with hypothyroidism and fibromyalgia by analyzing the temperature of the brown adipose tissue (BAT). A total of 166 individuals from 1000 thermographic electronic medical records were classified into four groups: Group HP + FM-50 individuals with hypothyroidism and fibromyalgia; Group FM-56 individuals with fibromyalgia only; Group HP-30 individuals with hypothyroidism only, and Group Control-30 healthy individuals. The thermal images from the electronic medical records were acquired by a FLIR T650SC infrared camera (used for thermometry) and the temperature data for each group were statistically analyzed. Group HP + FM showed *r* = 0, meaning that the average temperatures of the thyroid and BAT are independent of each other. Groups FM, HP and Control showed *r* = 1, meaning that the average temperatures of the thyroid and BAT were directly related. Our findings showed that the average temperatures of the thyroid and BAT regions are similar. Also, there was no correlation between thyroid gland temperature and the presence of hypothyroidism or fibromyalgia using thermometry.

## Introduction

Medical infrared thermography (MIT) is a noninvasive and nonradioactive analysis method capable of analyzing physiological functions related to the control of skin temperature, an important organ for controlling body temperature^1,2^. This thermography´s technique allows the evaluation of physiological changes^2,3^, with applications in the field of medicine to identify neurological, rheumatological and dermatological disorders, vascular diseases, urologic, and gynecological and orthopedic pathologies^3–7^, and this method can provide support for sports medicine^3^.

Fibromyalgia (FM) is a rheumatologic disorder, of undefined cause, but its development is associated with the central nervous system’s regulation of pain^8,9^, neurosensory, neuroendocrine and neurotransmitter-related disorders, as well as a genetic predisposition^10,11^. FM is characterized by musculoskeletal pain in diffuse to chronic intensity and is associated with symptoms such as fatigue, sleep disturbances, palpebral venous congestion, morning stiffness, diffuse paresthesia, subjective sensation of edema, cognitive disorders, depression and anxiety^10,12,13^. When a thermography examination is performed in a patient with FM, the patient presents a characteristic image pattern of the mantle sign that means a wide and diffuse hyper-radiation on the cervicothoracic region, low level cooling of the extremities due to the Raynaud phenomenon^11,14,15^, and hyperperiocular radiation, resulting from palpebral venous congestion due to non-repairing sleep and fatigue (daytime tiredness)^11,13,16,17^.

MIT can quantify the non-shivering thermogenesis (NST) of brown adipose tissue (BAT)^16^. BAT is an endocrine adipose tissue with attributes to dissipate energy as heat in response to changes in temperature and diet^18^. It is an important regulator of energy balance and metabolism in homeothermic animals and it is metabolically less active in adults than in newborns, because its main function is thermogenesis, which is the ability to burn calories to generate heat^7,15,18^. BAT can be located deep within the neck and trunk, near the great vessels, in the supraclavicular, supra-axial, paraspinal, and perirenal regions, in sympathetic ganglia and striations of the skeletal muscles and, finally, just below the skin^7,15,18^. Resting BAT activity and FM incidence are higher in women^15,19–21^, with lower adaptive thermogenesis in this group^7^.

BAT plays an integral role in adaptive thermogenesis due to its ability to rapidly generate significant amounts of heat from fatty acids and glucose, allowing the dissociation of ATP production from the mitochondrial breathing. As heat is lost from the body, this represents the liquid loss of energy and has the potential to contribute to body weight^22–27^. The interaction between BAT activation and the thyroid is complex. The thyroid, as an important regulator of energy expenditure, can modulate the heat generation capacity of BAT, thus, the thyroid hormone reduced concentrations can therefore affect BAT activity directly or centrally reduce sympathetic nervous system (SNS) activation^22^.

BAT’s thermogenic activity is increased by the same conditions that aggravate FM symptoms due to the distribution of brown adipose enervation and surrounding tissues^7,28,29^. In addition, when there is a decrease in body temperature, there is also a decrease in metabolic and body temperature rates^15,30,31^, because BAT distribution is related to tender points (TP), it is thought that it may become sensitized and cause pain in the TP region^15,16,29,30^. Stress and cold stimulate thermogenesis and aggravate FM symptoms, therefore, patients feel cold intolerance. Warming temporarily suspends thermogenesis and pain, and heat suspends thermogenesis and relieves FM symptoms^5,16,30^.

Hypothyroidism (HP) is a disorder that occurs when the thyroid gland does not produce enough thyroid hormone to meet the body's needs to regulate metabolism, that is, the way the body uses energy^32,33^; and affects almost every organ in the body. The most prevalent form is caused by a failure of the gland itself, but also, hypothyroidism may occur due to hypothalamic or pituitary disease^33,34^**.** Hypothyroidism (HP) may exhibit nonspecific symptoms similar to FM, such as fatigue, sleep disorders, intestinal changes, weight gain and body aches^33^. Compared to a 1–5% incidence of hypothyroidism in the general population, there is a reported incidence of 10–14% in patients with FM^35,36^.

Thus, an incidence of hypothyroidism in FM shows a positive correlation between the TSH level and pain distribution, that is, it increases the possibility that FM pain distribution is associated with hypothyroidism^37,38^. And there is still evidence that most cases of FM are associated with difficulties in the production or use of the thyroid, although they have documented the similar appearance of FM and HP^39,40^. It is believed that there is a greater prevalence of thyroid problems in FM patients, but it is difficult to confirm if treatment of these conditions will also improve FM symptoms. Under the condition of no pathology/disorder, the thyroid gland has the same temperature as the adjacent soft tissues. However, in the presence of a hypermetabolism of the nodules or the whole gland, it will be highlighted in the thermogram. In both cases it will be possible to assess the temperature of the region using standard measurement points in the thermographic images^11^.

Based on the above arguments, the following research question was raised: is the noninvasive MIT technique effective in assessing thyroid gland temperature in HP and FM patients?

It is known that the symptoms of FM are similar to those of HP. Thus, in certain situations patients are misdiagnosed since FM diagnosis is essentially clinical and HP is laboratory based. An investigation of FM, HP and a control group (healthy individuals) can help determine whether there is a connection between the thyroid gland metabolism level in these groups. This investigation can help determine if, within the selected database, there is a thermographic correlation in individuals with or without HP. Therefore, the objective of this work was to investigate the thermal changes in the thyroid gland and the BAT regions of patients with HP and FM to evaluate if there was a correlation between these regions’ temperatures in patients with these diseases compared to a control group (without HP and FM).

## Materials and methods

This study was approved by the Research Ethics Committee (CEP) of the Federal University of Technology – Paraná (UTFPR) via Plataforma Brasil, protocol number 1.054.356, according to the Brazilian Ministry of Health rules that follow all International Ethical Guidelines for Biomedical Research Involving Human Subjects, produced by the Council for International Organizations of Medical Sciences (CIOMS).

As the research have been conducted using a database, without any interview with patients, the need for informed consent was waived by the Ethics Committee of the Federal University of Technology- Parana (UTFPR).

The research work was carried out from January 2017 to December 2019 and, relating to the objectives, the study design was exploratory retrospective and the approach was quantitative transversal. The data acquisition process was based on access to a database of a thermography clinic, where the records of patients diagnosed with fibromyalgia, hypothyroidism and without both diseases were selected. The electronic records of 166 individuals from a total of 1000 records were selected using the following inclusion criteria:

- Individuals attended the clinic from 2014 to 2016;
- The data was collected retrospectively;
- Records contained anteroposterior (AP) upper orthostatic and AP cervical extension images;
- Complete thermographic reports;
- Male and female individuals;
- Over 18 years old;
- The patients follow the standardized exam preparation recommendations of the Brazilian Society of Thermology—ABRATERM and, according to the rules of the thermography clinic, patients should be fasting for at least 2 h before the exams;
- All patients remained for 15 min in the laboratory for acclimatization before the exams;
- The body mass index (BMI) was not considered in the survey because the data was not available in the database fields;
- Groups with FM and HP, FM only, HP only and healthy individuals;
- Laboratory reports for HP diagnosis. All patients had their serum TSH levels controlled;
- Radiological examination reports;
- Demographics of the patient´s profile;
- FM diagnosed according to the criteria of the American College of Rheumatology (ACR)^13,38^, and HP diagnosed by laboratory examination; and
- Preliminary questionnaire for FM criteria that assists the medical professionals in the diagnosis (ACR/2010)^13,40^. The results obtained within this questionnaire for the generalized index of pain (WPI), and scale of gravity of symptoms (SS) should remain between WPI ≥ 7/19 + SS ≥ 5 or WPI 3–6 + SS ≥ 9.

The individuals’ records were selected from the database and classified into four groups:

- Group HP + FM: patients with FM and HP, 50 patients, 48.4 ± 13.6 years old, 48 females and 2 males, WPI = 11.6 and SS = 9.4;
- Group FM: patients with FM only, 56 patients, 44.8 ± 8.7 years old, 48 females and 8 males, WPI = 10.4 and SS = 8.8;
- Group HP: patients with HP only, 30 patients, 24 females and 6 males, 53.0 ± 14.0 years old, WPI = 3.5 and SS = 3.5.
- Group Control: control group, with 30 healthy individuals, 44.5 ± 11.0 years old, 18 females and 12 males, WPI = 3.8 and SS = 5.3.

**Figure 1:**
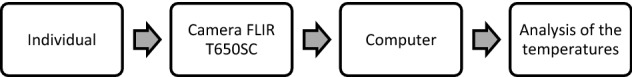
Setup of a thermal data measurement system.

The database thermal images were acquired retrospectively using the FLIR T650SC infrared Camera (Flir Systems Inc. Nashua, NH, USA) with the technical specifications shown in Table 1. The AP thermography’s images with cervical extension and the AP upper orthostatic images were obtained with individuals positioned at 1 m from the camera in an orthostatic position. The images were processed on a computer using the FLIR Report program (FLIR Tools, version 4.1.140661001) and the temperature data were obtained and analyzed. The block diagram of the setup used to acquire the thermal images is shown in Fig. 1.

**Table 1:** Technical specifications of the FLIR T650SC infrared camera used to acquire the patients’ thermal images.

Camera parameters	Values
Resolution	640 × 480 pixels
Thermal Sensitivity (at 30 °C)	< 20 mk @ 30 °C
Field of view	25° × 19°/0.25 mm
Image frequency	30 Hz
Spectral range	7.5 to 14 μm
Spatial resolution	0.68 mrad
Focus	Continuous, one chot or manual
Temperature range	− 40 °C to + 150 °C; + 100 °C to + 650 °C; + 300 °C to 2000 °C
Measurement accuracy	± 1 °C or ± 1% of reading
Temperature accuracy	± 2 °C
Temperature resolution	0.1 °C
Emissivity	0.98
Reflection temperature	20 °C
Atmospheric temperature	23 °C
Relative humidity	50%
Distance from camera to the subject	1 m

**Figure 2:**
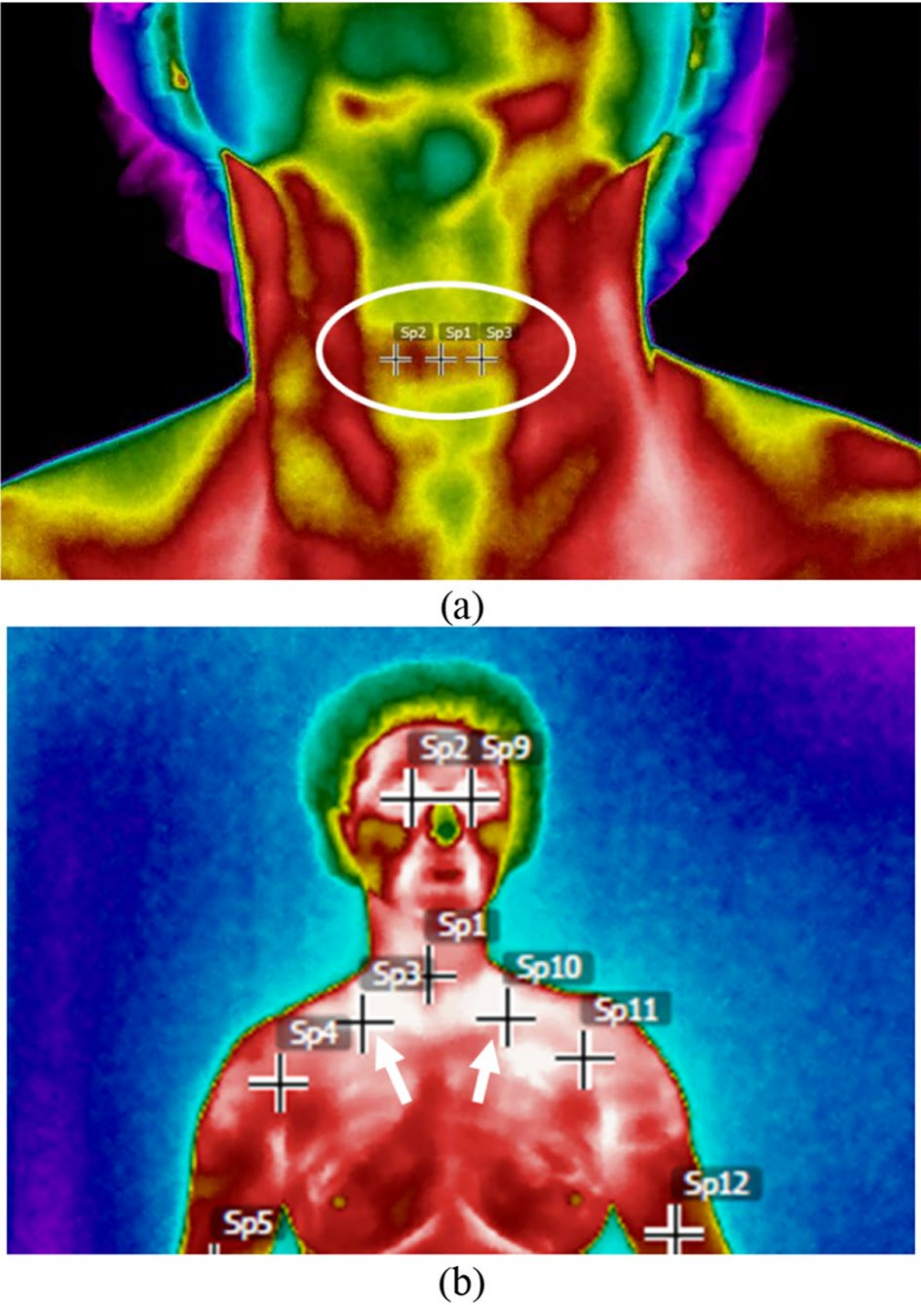
Points to measure the temperature in the (**a**) thyroid region and (**b**) in the brown adipose tissue (BAT) region (SP3 and SP10).

The AP thermography’s images with cervical extension were analyzed using the average temperatures of three thermal points (Fig. 2a) selected by the researchers. For the AP upper orthostatic images, the thermal points located bilaterally (SP3 and SP10) in the medial supraclavicular region were used, that is, in the BAT region (Fig. 2b).

With the temperatures obtained for each individual, the data were organized into a table with the following specifications: data were organized with a numerical identification for each individual, with an associating age, gender, and FM diagnosis according to the criteria of the ACR/2010 and Preliminary Questionnaire for FM Criteria^15^; a diagnosis for HP (based on clinical laboratory tests and anamnesis analysis), and temperatures of the thyroid gland and BAT regions.

**Figure 3:**
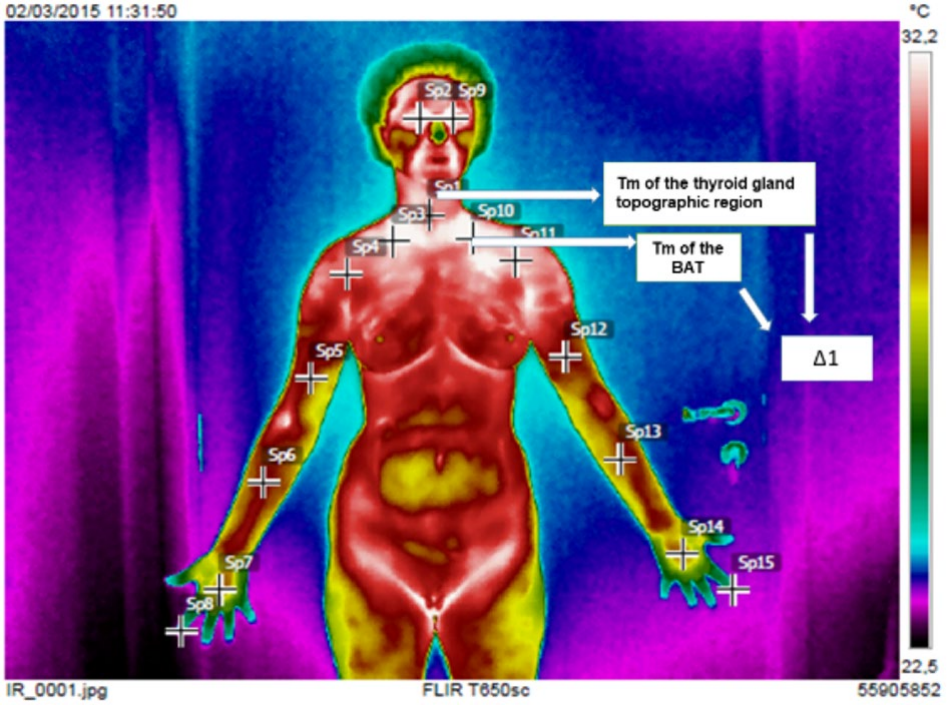
Points used to calculate the average temperature difference (Δ1) between the Tm of thyroid region and BAT.

Temperatures obtained for each group were statistically analyzed with the Microcal Origin 6.0 software (OriginLab Corporation, Northampton, Massachusetts, USA), which obtained the average age and standard deviation of the groups, the average temperature (Tm) and standard deviation for each group (thyroid and BAT), comparisons between the groups, and the average temperature difference (Δ1) between each region studied (Fig. 3). The software also applied a variance test (ANOVA) to evaluate if the average temperatures of the two regions were equal or different. Student’s t-test was used to compare the average temperature differences between the groups.

The Action Stat application was used to obtain the Principal Component Analysis (PCA). Individuals were grouped according to their variances, that is, according to their behavior within the population, represented by the variation of their set of characteristics (Thyroid and BAT temperatures). The analysis allowed for a reduction in the number of variables to provide a view of the data set, and thereby helped to identify the most important variables in the space of the main components.

The last statistical analysis was used to obtain the Pearson’s linear correlation coefficient or "Pearson's *r*" to assess the degree of correlation and the direction of this correlation. Thus, if the correlation the variables is equal to 1, it is a positive correlation, meaning the variables are directly proportional to each other. If the correlation is negative (*r* =  − 1), the variables are indirectly proportional to each other. Finally, if *r* = 0, the two variables are not linearly dependent.

### Ethics approval

This study was approved by the Research Ethics Committee (CEP) of the Federal University of Technology – Paraná (UTFPR) via Plataforma Brasil, protocol number 1.054.356, according to the Brazilian Ministry of Health rules that follow all International Ethical Guidelines for Biomedical Research Involving Human Subjects, produced by the Council for International Organizations of Medical Sciences (CIOMS).

### Consent to participate

As the research was carried out in a database, without conducting an interview with patients, the need for informed consent was waived by the Ethics Committee of the Federal University of Technology of Paraná (UTFPR).

## Results

From the 166 individuals selected for this research, 106 were diagnosed with FM according to the ACR criteria^13,40^, 80 patients were diagnosed with HP, and 30 showed no evidence of the pathology.

**Figure 4:**
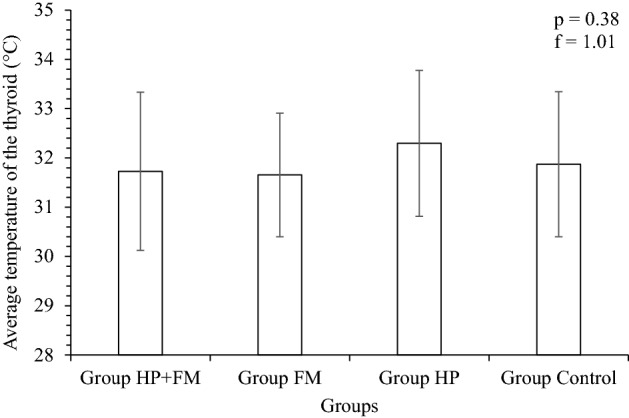
Average temperatures of the thyroid gland region for Group HP + FM, Group FM, Group HP and Group Control.

The temperature difference found in the thyroid gland region of the individuals in Group HP + FM was + 1.1 °C. The average temperatures (Tm) of the three selected points in the thyroid gland were 31.7 ± 1.6 °C for Group HP + FM, 31.6 ± 1.2 °C for Group FM, 32.3 ± 1.5 °C for Group HP and 31.9 ± 1.5 °C for Group Control (see Fig. 4). The obtained differences Δ1 were: 0.1 °C between groups FM + HP and FM, 0.6 °C between groups FM + HP and HP, 0.2 °C between groups FM + HP and Control, 0.7 °C between groups FM and HP, 0.3 °C between groups FM and Control, and 0.4 °C between groups HP and Control.

The variance analysis showed that the samples are not significantly different from each other, with an occurrence probability (*p*) of 0.38 and a frequency of occurrence (f) of 1.01.

**Figure 5:**
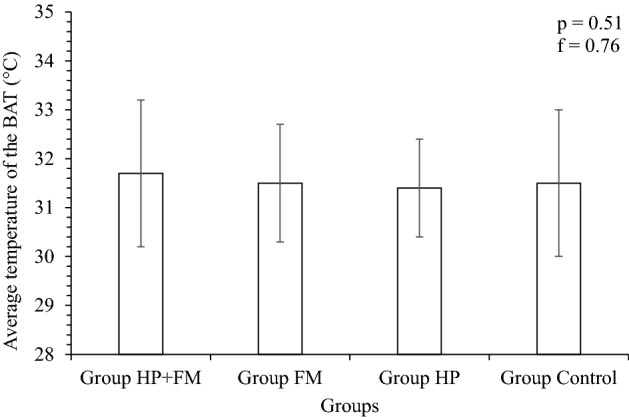
Average BAT temperatures for Group HP + FM, Group FM, Group HP and Group Control.

The average temperature of the two points (SP3 and SP10) in the BAT region was 31.7 ± 1.5 °C for Group HP + FM, 31.5 ± 1.2 °C for Group FM, 31.4 ± 1.0 °C for Group HP and 31.5 ± 1.5 °C for Group Control (see Fig. 4). The Δ1 was 0.2 °C between groups FM + HP and FM, 0.3 °C between groups FM + HP and HP, 0.2 °C between groups FM + HP and Control, 0.1 °C between groups FM and HP, 0.0 °C between groups FM and Control, and 0.1 °C between groups HP and Control (see Fig. 5). The analysis of variance showed that the samples are not significantly different from each other, with *p* = 0.51 and f = 0.76.

Comparing the average temperatures of the thyroid gland region with the average temperatures of the BAT, the Pearson’s correlation coefficient "*r*" was 0 (zero) for the overall analysis, where all groups were compared with each other. The Pearson’s coefficients were: *r* = 0 for Group HP + FM, *r* = 1 for Group FM, *r* = 1 for Group HP, and *r* = 1 for Group Control. The correlation equal to 1 means that the variables have a positive correlation and are directly proportional.

The ANOVA test, when applied to test for a comparison between the average temperatures of the thyroid and BAT, showed that the mean temperatures are not significantly different, and the t-test showed that the samples are not significantly different. The obtained results were f = 1.4 and *p* = 0.24 for Group HP + FM, *p* = 0.35 and f = 0.55 for Group FM, f = 3.0 and *p* = 0.08 for Group HP, and f = 0.40 and *p* = 0.71 for Group Control.

The principal component analysis (PCA) for the average temperatures of the thyroid gland region and BAT for Group HP + FM showed that the first principal component (PC1) explains 89.7% of the total variation, where average temperatures were grouped according to their variance (0.89). PC2 explained 10.3%, with a variance of 0.10. The PC1 for Group FM explains 92.5% of the total variation, with a variance of 0.92, and PC2 explains 7.5% with variance of 0.07. The PC1 for Group HP explains 55.3% of the total variation of the samples with a variance of 0.74, and PC2 explains 44.6% with a variance 0.69. The PC1 for Group Control explains 96.7% of the total variation of the samples with a variance of 0.96, and PC2 explains 3.3% with variances of 0.03.

**Figure 6:**
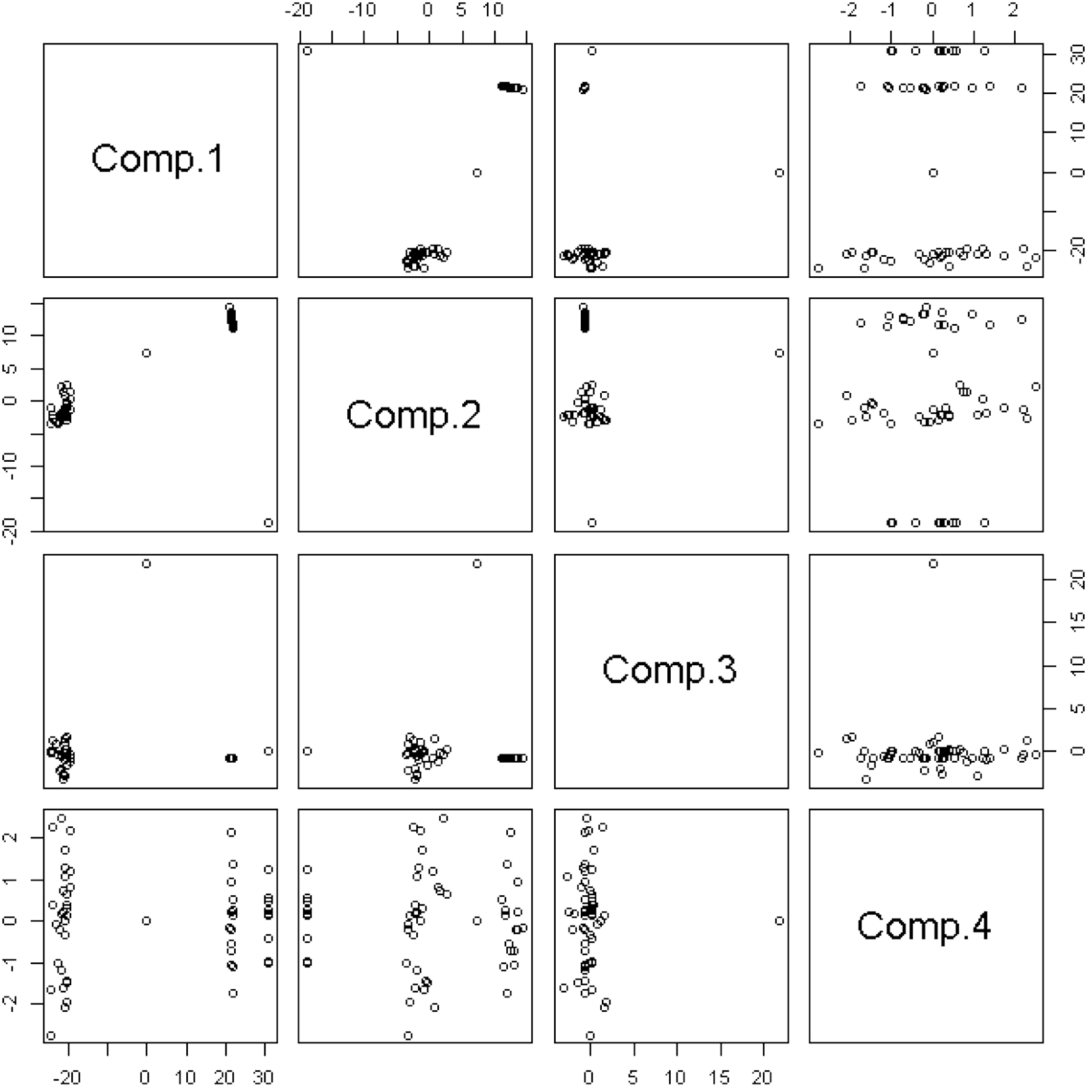
PCA analysis for Group HP + FM, Group FM, Group HP and Group Control.

Analyzing the four groups, the first principal component (PC1) explains 42.96% of the total variation and, according to the eigenvectors, the weights variations of Group HP + FM are negatively high for this component. That is, the higher the average temperatures of the thyroid gland region and BAT were, the lower the score of the first component was. For Group FM, Group HP and Group Control, according to the eigenvectors, the weights variations are positive. Therefore, with an increase in temperature, the score of the first component will also increase (Fig. 6).

## Discussion

The temperature difference found in the thyroid region of patients with FM and HP (Group 1) by thermography was + 1.1 °C with *r* = 0, so the temperature variation does not impact in average temperatures of this group and the variables had no correlation. That is, for this study group, thyroid metabolism variation and BAT temperature variation are independent of each other, independent of the patient´s diagnosis for HP and FM. However, it was found by other authors that average temperature values higher than 0.26 °C from normal human temperature (36.5 °C to 37.0 °C) already show alterations in thyroid gland metabolism^41^. It should be noted that in the study, the authors evaluated only the temperature variation of the thyroid gland, without correlating another variable as considered in this paper such as HP and FM.

The average temperatures of the three regions of the thyroid gland were similar to each other, as the analysis of variance and Δ1 between groups ranged from 0.1 to 0.7 °C. According to some results reported in the literature for the trunk region, temperature variations of 0.17 ± 0.042 °C showed abnormalities^38^, which do not agree with the results of other research conducted in the same anatomical region that found temperature variations of 0.5 °C to 1 °C^41^. The patients had homogeneous characteristics, besides, TSH serum levels controlled.

HP is among the thyroid dysfunctions that have nonspecific symptoms similar to symptoms of FM^33,42^. In the literature it can be found that HP dysfunction presents alterations in the hypophysis-hypothalamic axis where FM patients have less thyrotropin (TSH)^33,43–50^. Therefore, in FM patients, pain is being investigated related to changes in the hypothalamic-hypophysis-adrenal axis, and it is still unknown if both cases of HP and FM hyperactivity are present in these axes. It is not known whether this hyperactivity is genetically based or whether it is a result of stress throughout life, or in a specific situation.

BAT is also a target of thyroid hormones, where they present a large number of 3, 5, 3′-triiodothyronine (T3) receptors that are 70% occupied at room temperature and approximately 100% occupied during cold exposure^45,51^. In fact, BAT has its own T3-generating mechanism, due to the local activity of the enzyme deiodinase type II, D2. The activity of this enzyme and T3 concentration increases 3 to 50 times during the sympathetic activation of BAT, resulting in an increase in the local impact of T3, without affecting the plasma concentration of T3. Thus, sympathetic activation of BAT results in local hyperthyroidism and increased heat production^45,51^. In contrast, this study analyzed hypothyroidism patients, who presented low metabolic indices, cold intolerance, muscle weakness, fatigue, bradycardia, myxedema, depression and cognitive deficits. It was observed that the average temperatures of BAT showed only a difference of 0.2 °C from Group HP + FM (31.7 °C) to Group FM (31.5 °C), and 0.3 °C from Group HP + FM (31.7 °C) to Group HP (31.4 °C), 0.2 °C between groups FM + HP (31.7 °C) and Control (31.5 °C), 0.1 °C between groups FM (31.5 °C) and HP (31.4 °C), 0.0 °C between groups FM (31.5 °C) and Control (31.5 °C), and 0.1 °C between groups HP (31.4 °C) and Control (31.5 °C). Thermal imagens have been established as a valid alternative for diagnosis for BAT activity and FM^5,22^. In this research, small differences in temperatures were found between the groups, as the thermography equipment measures the body's infrared energy, demonstrating the thermal distribution of the skin surface by a high-resolution image. In this way, the temperature can be measured from the energy emitted by the skin surface in a totally safe way, that is, without any contraindications^11^.

Nevertheless, according to the results, there were no significant differences in the temperatures of BAT between the three groups, and *r* = 1, reinforcing that the average temperatures of the thyroid region and BAT are interdependent. That is, when there is a variation in the metabolic rate of the thyroid, there will be a variation in the rate of BAT activity. Therefore, elucidating this result requires knowing the impacts of thyroid hormones in human biology, in which acceleration of energy metabolism and ATP turnover are related as a result of energy transformation to heat production^52^. In these terms, thyroid hormones as mediators of homeotherm are present in homeothermic animals, and are capable of stimulating heat production. In humans with HP there is hypothermia and cold intolerance, lose part of their homeothermy and the ability to adapt to the environment^45,52^.

In the literature there are indications that other diseases should be excluded before starting FM treatment, because there are similarities of symptoms^33^ to other diseases such as HP, hyperparathyroidism (parathyroid adenoma) and autoimmune thyroiditis due to autoimmune disease^46,53,54^. Therefore, it was necessary to know the patient’s profile from the selected records to classify groups and to discard other diseases that could impact the results. Thus, the following symptoms were observed in the questionnaire for preliminary FM criteria: chronic pain in the body, showing hyper-radiant regions^54–57^, depression, tiredness, non-repairing sleep, signs of periocular congestion, constipation, diarrhea, headache and the correlation of chronic generalized pain such as myalgias, arthritis, arthrosis and rheumatism^33,45,49^. All patients maintained controlled treatment for thyroid dysfunction, especially for HP.

According to the parameters of the questionnaire for preliminary FM criteria, a patient with FM is diagnosed as such if he or she presents a composition of factors such as the combination between a generalized index of pain (WPI) ≥ 7/19 + a scale of gravity of symptoms (SS ≥ 5) and/or WPI between 3 and 6 + SS ≥ 9, besides clinical exams^38,53,54,58,59^.

However, patients selected for this study had, in Group HP + FM, a mean WPI = 11.6 and a mean SS = 9.4, that in combination confirmed the disease. Group FM had a mean WPI = 10.4 and a mean SS = 8.8, also confirming FM but with normal HP laboratory results. Group HP had a mean WPI = 3.5 and a mean SS = 3.5, the patients included in this group showed no FM, and Group Control presented a mean WPI = 3.8 and a mean SS = 5.3; confirming normality for FM according to ACR^38^.

In this research, it was observed that FM patients presented mantle signals that could be indicated of neurovegetative disorders, besides periocular congestion (nonrestorative sleep), vasospasms in the extremities and other signs^30,52^.

Results of the thermography diagnostic examination, although not a definitive diagnosis, suggest that mantle phenomena together with peripheral vasoconstriction can support clinical diagnosis and play important roles in the follow-up of FM patients as markers of neurovegetative dysfunction present in the disease^54^.

This study followed the outlined objective using a database containing thermographic images and, to carry out the research, there was no contact with the patients. The database with the medical records did not include some data, such as the BMI and serum TSH levels. Future works can be done by analyzing other variables, such as temperatures of other parts of the body, other pathologies, as well as analyzing the impact of BMI.

## Conclusions

Analyzing the results in this work, it was possible to conclude that the average temperatures of the three thyroid points (*p* = 0.38, f = 1.01) and the two BAT points (*p* = 0.51, f = 0.76) for the surveyed groups were not different, with a Pearson’s correlation coefficient (*r*) equal to zero, signifying that thyroid metabolism variation and BAT temperature variation are independent of each other, independent of the patient´s diagnosis for HP and FM. A similar result was confirmed when applying the Pearson correlation coefficient between the data obtained from the thyroid gland and the BAT regions. The group composed by patients with HP and FM, showed *r* = 0, meaning also that the thyroid and BAT temperatures are not correlated. Therefore, a variation in the metabolic activity of the thyroid gland does not imply BAT activity.

As for the group composed of individuals with FM and without HP, the group for individuals with HP, and the control group (healthy subjects), it was found *r* = 1, meaning that the average temperatures of the thyroid and BAT are directly related, where a variation in the metabolic activity of the thyroid gland exhibits an interaction with the variation in the metabolic rate of BAT.

Thus, it was possible to conclude that this thermometry technique can be used for measuring the temperatures of the thyroid gland and BAT regions in order to evaluate their changes. This study has shown that that mean thyroid and BAT temperatures are similar and there was no correlation between thyroid temperature and the presence of hypothyroidism or fibromyalgia using thermometry.

## Data Availability

Not applicable.
